# Dipeptide Repeat Pathology in *C9orf72*-ALS Is Associated with Redox, Mitochondrial and NRF2 Pathway Imbalance

**DOI:** 10.3390/antiox11101897

**Published:** 2022-09-25

**Authors:** José Jiménez-Villegas, Janine Kirby, Ana Mata, Susana Cadenas, Martin R. Turner, Andrea Malaspina, Pamela J. Shaw, Antonio Cuadrado, Ana I. Rojo

**Affiliations:** 1 Department of Biochemistry, Medical College, Autonomous University of Madrid (UAM), 28029 Madrid, Spain; 2 Instituto de Investigaciones Biomédicas “Alberto Sols” (CSIC/UAM), 28029 Madrid, Spain; 3 Instituto de Investigación Sanitaria La Paz (IdiPaz), 28029 Madrid, Spain; 4 Centro de Investigación Biomédica en Red de Enfermedades Neurodegenerativas (CIBERNED), 28029 Madrid, Spain; 5 Sheffield Institute for Translational Neuroscience, University of Sheffield, Sheffield S10 2HQ, UK; 6 Centro de Biología Molecular “Severo Ochoa” (CSIC/UAM), 28049 Madrid, Spain; 7 Instituto de Investigación Sanitaria Princesa (IIS-IP), 28006 Madrid, Spain; 8 Nuffield Department of Clinical Neurosciences, University of Oxford, Oxford OX3 9DU, UK; 9 Neuroscience and Trauma Centre, Blizard Institute, Barts and The London School of Medicine & Dentistry, Queen Mary University of London, London E1 2AT, UK; 10 Queen Square Motor Neuron Disease Centre, Neuromuscular Department, Institute of Neurology, University College London, London WC1N 3BG, UK

**Keywords:** NRF2, amyotrophic lateral sclerosis, *C9orf72*, dipeptide repeat proteins

## Abstract

The hexanucleotide expansion of the *C9orf72* gene is found in 40% of familial amyotrophic lateral sclerosis (ALS) patients. This genetic alteration has been connected with impaired management of reactive oxygen species. In this study, we conducted targeted transcriptional profiling in leukocytes from *C9orf72* patients and control subjects by examining the mRNA levels of 84 redox-related genes. The expression of ten redox genes was altered in samples from *C9orf72* ALS patients compared to healthy controls. Considering that Nuclear factor erythroid 2-Related Factor 2 (NRF2) modulates the expression of a wide range of redox genes, we further investigated its status on an in vitro model of dipeptide repeat (DPR) toxicity. This model mimics the gain of function, toxic mechanisms attributed to *C9orf72* pathology. We found that exposure to DPRs increased superoxide levels and reduced mitochondrial potential as well as cell survival. Importantly, cells overexpressing DPRs exhibited reduced protein levels of NRF2 and its target genes upon inhibition of the proteasome or its canonical repressor, the E3 ligase adapter KEAP1. However, NRF2 activation was sufficient to recover cell viability and redox homeostasis. This study identifies NRF2 as a putative target in precision medicine for the therapy of ALS patients harboring *C9orf72* expansion repeats.

## 1. Introduction

The hexanucleotide expansion located in the first intron of the *C9orf72* gene affects 40% of familial amyotrophic lateral sclerosis (ALS) patients [1] and causes a devastating disease that involves the progressive degeneration of motor neurons and muscle denervation [2]. Current therapies for ALS, riluzole [3] and edaravone [4], have produced limited clinical benefit [5]. It is imperative to elucidate the primary mechanisms responsible for motor neuron loss in order to design effective therapeutics against the toxicity derived from *C9orf72* expansion. 

Haploinsufficiency has been proposed as a toxic mechanism induced by (G_4_C_2_)_n_ expansion [6]; however, *C9orf72* null mice do not develop motor neuron dysfunction, suggesting the existence of other mechanisms involved in disease pathophysiology [7]. Two possible gain of function mechanisms have been highlighted, including the production of long repeat RNA and dipeptide repeat proteins (DPRs). DPRs are translated from long repeat RNAs by a non-canonical process, repeat associated-non-AUG (RAN) translation, giving rise to five different species. Poly-GR, poly-GA and poly-GP are produced from the sense strand and poly-PR, poly-GP and poly-PA from the antisense strand [8,9]. Of these, poly-GA, poly-GR, and poly-PR have shown toxicity in cellular models, and these arginine-containing DPRs (Arg-DPRs) are the most toxic [10,11,12]. 

Emerging evidence suggests that oxidative stress and mitochondrial dysfunction are important elements of mutant *C9orf72* pathology [13,14]. A central regulator of the antioxidant response in cells is the transcription factor NRF2 (Nuclear factor erythroid 2-Related Factor 2). NRF2 is principally controlled by regulation at the protein level by the ubiquitin–proteasome system due to the presence of at least two degradation domains, exhibiting a half-life of approximately 30 min depending on the cell type [15,16]. The best-characterized regulatory mechanism involves the E3 ubiquitin ligase adapter KEAP1 (Kelch-like-ECH-associated protein 1) which targets NRF2 for ubiquitin/proteasome degradation [17]. Oxidative stress or electrophilic molecules modify several redox-sensitive cysteines in KEAP1 [18,19] in a way that renders it no longer effective in connecting NRF2 with the ubiquitination machinery [20]. Thus, newly synthesized NRF2 escapes degradation, accumulates in the nucleus, and activates its cytoprotective program through the activation of genes containing NRF2-regulated Antioxidant Response Elements (ARE). The repertoire of NRF2-regulated genes includes genes related to redox homeostasis (HMOX1, NQO1, GCLM, GCL, GPX), proteostasis control (PSMB7, ULK1, SQSTM1, LAMP2A) and inflammatory response (IL1β, IL6, MARCO) [21], which are processes thought to be dysregulated in C9orf72 patients. However, the pathogenic role for DPRs in neurotoxicity related to the regulation of the endogenous antioxidant defense remains poorly understood.

In this study, we sought to determine the alterations in redox gene expression in *C9orf72*-ALS patients and the interplay between arginine containing DPRs, control of redox alterations, and NRF2. Our results suggest NRF2 as a candidate therapeutic target for *C9orf72*-ALS patients.

## 2. Materials and Methods

### 2.1. Targeted qRT-PCR Analysis in Human Samples

The case-control study was performed on 7 *C9orf72* mutation carriers and 7 healthy controls who were recruited according to the criteria specified by the UK ‘AMBRoSIA’ ALS biomarker cohort study. *C9orf72* expansion testing was carried out as described in [22]. Venous blood was collected in EDTA-containing tubes for leukocyte RNA isolation. Within 15 min of venipuncture, an EDTA blood bottle was passed through an RNA extraction kit LeukoLOCK™ filter from LeukoLOCK™ Total RNA Isolation System (Invitrogen, Waltham, MA, USA; ID AM1923)). A total of 3 mL of phosphate buffered saline was then passed through the filter, followed by 3 mL of the RNAlater™ solution (Invitrogen, Waltham, MA, USA; ID AM7021). Leukocyte RNA which was bound to the filter was isolated according to the manufacturer instructions and stored at −80 °C until further processing. 

RNA integrity was assessed using an RNA 6000 Nano Kit (Agilent, Santa Clara, CA, USA; ID 5067-1511), according to manufacturer’s instructions. Briefly, 1 μL of each sample (approximately 200 ng of RNA) was loaded over the Gel-Dye Mix placed previously in the chip wells. Electrophoresis, data analysis and calculation of the RNA integrity number (RIN) were achieved by employing an Agilent 2100 Bioanalyzer (Agilent, Santa Clara, CA, USA). *C9orf72* carriers were matched to healthy controls of a similar age, gender and whose samples had similar RNA integrity values (Table 1).

Reverse transcription using 400 ng of RNA from each control or C9*orf*72 patient sample was performed with an RT2 First Strand Kit (Qiagen, Hilden, Germany; ID 330401)) according to the manufacturer’s instructions. The expression of 84 key genes involved in oxidative stress/antioxidant response was analyzed with the RT2 Profiler™ PCR Array Human Oxidative Stress Plus (Qiagen, Hilden, Germany; ID PAHS-065Y)) for each sample. Data from the 84 genes can be found in Supplementary Table S1. The SYBR Green chemistry of the Applied Biosystems 7900 HT FAST Real-Time PCR system (Applied Biosystems, Waltham, MA, USA) was used. The expression level of each gene was normalized with the geometric mean of the housekeeping genes ACTB, B2M, GAPDH, HPRT1, RPLP0 to produce the ΔCt value and this was further normalized with the mean of the control group to eventually calculate 2^−ΔΔCt^ values or the fold of change (FC). Data are represented and statistical analysis was performed with log2(FC). Results are presented as the mean ± standard error of the mean (SEM) from each experimental group. Statistical analysis was performed using a script of the R programming language. Since data were not normally distributed (Shapiro–Wilk test, *p* < 0.05), the differences in gene expression between patients and controls were evaluated using the non-parametric Wilcoxon rank-sum test. The difference in gene expression was considered significant at *p* < 0.05.

### 2.2. Cell Culture and Reagents

The Mouse Motor Neuron-Like Hybrid Cell Line (NSC34) (Cedarlane Laboratories, Burlington, Canada) was grown in DMEM (Dulbecco’s modified Eagle’s medium with high glucose; Sigma-Aldrich, San Luis, MO, USA; ID D5648) supplemented with 10% heat inactivated fetal bovine serum (FBS, Biowest, Nuaillé, France), 2 mM glutamine (Gibco, Waltham, MA, USA; ID 25030081) and 80 µg/mL gentamicin (Normon Laboratories, Tres Cantos, Spain). NSC34-Sham and NSC34-(G_4_C_2_)_102_ are isogenic cells lines, as previously described [23]. These cells contain an Flp-In™ T-Rex™ expression cassette, encoding an FRT recombination site and a tetracycline repressor element, where (G_4_C_2_)_102_ and Sham sequences are stably integrated from pcDNA5/FRT/TO^®^ and pcDNA/FRT/TO^®^-(G_4_C_2_)_102_. NSC34-sham and NSC34-(G_4_C_2_)_102_ cells were grown in DMEM supplemented with 10% tetracycline-free tested FBS (Takara Bio, Kusatsu, Japan; ID 631106) which was previously heat inactivated, 2 mM glutamine (Gibco, Waltham, MA, USA; ID 25030081), 80 μg/mL gentamicin (Normon Laboratories, Tres Cantos, Spain), 100 μg/mL hygromycin B and 5 μg/mL blasticidin (InvivoGen, San Diego, CA, USA; IDs ant-hg-1 and ant-bl-1). For expression of the hexanucleotide repeat cassette, 1 μg/mL tetracycline (Gibco, Waltham, MA, USA; ID A39246) was added to the medium and refreshed every three days. 3-(4,5-dimethylthiazol-2-yl)-2,5-diphenyltetrazolium (MTT), dimethyl fumarate, actinomycin D and MG132 were purchased from Sigma Aldrich (San Luis, MO, US; IDs M5655, 242926, A9415, P8833, C2211). Dimethyl fumarate, MG132 and actinomycin D were diluted in dimethyl sulfoxide. Puromycin was diluted in H_2_O. HA, HA-GR_20_ and HA-PR_20_ were synthetized by GenicBio Limited (Shangai, China), diluted in H_2_O, and kept as 1 mM stocks at −70 °C.

### 2.3. Transfection, Lentiviral Production and Transduction

NSC34 cells were transfected with pEGFP-C3, pEGFP-GR_50_ and pEGFP-PR_50_ (kindly provided by Dr. JP Taylor, St. Jude Children’s Research Hospital. Memphis, TN, USA) or TK-Renilla (Promega, Madison, CA, USA) and ARE-LUC (Dr. J. Alam, Ochsner Clinic Foundation, New Orleans, LA, USA) using Lipofectamine 2000 (Invitrogen, Waltham, MA, USA; ID 11668019) in OptiMEM (Gibco, Waltham, MA, USA; ID 31985062) at a ratio of 1:1 (µL lipofectamine: µg DNA) according to the manufacturer’s instructions. EGFP expression was checked using a fluorescence microscope, ensuring that the transfection efficiency was greater than 70%. Pseudotyped lentiviral vectors were produced in HEK293T cells that were grown in DMEM - high glucose (Dulbecco’s modified Eagle’s medium with high glucose; Sigma-Aldrich, San Luis, MO, USA; ID D5648) supplemented with 10% heat inactivated fetal bovine serum (FBS; Biowest, Nuaillé, France), 2 mM glutamine (Gibco, Waltham, MA, USA, ID 25030081) and 80 µg/mL gentamicin (Normon Laboratories, Tres Cantos, Spain). HEK293T cells were transiently co-transfected with 10 μg of control or NRF2^ΔETGE^-V5 transfer vectors (after cloning into pWPXL lentiviral expression plasmid, (Addgene, ID 12257; deposited by Dr. Didier Trono, École Polytechnique Fédérale de Lausanne, Lausanne, Switzerland), 6 μg of the packaging plasmid pSPAX2 (Addgene, ID 12260) and 6 μg of the VSV-G envelope protein plasmid pMD2G (Addgene, ID 12259; deposited by Dr. Didier Trono) using Lipofectamine reagent and Plus^TM^ Reagent (Invitrogen, Waltham, MA, USA; IDs 18324012 and 11514015) according to the manufacturer’s instructions. For lentiviral transduction, NSC34 cells were grown to confluence and then transduced with supernatant from HEK293T-producing lentiviruses plus 10 μg/mL polybrene (Sigma-Aldrich, San Luis, MO, USA; ID TR-1003). The medium was removed 24 h after and expression of the proteins took place during 48 h.

### 2.4. Immunofluorescence Analysis

NSC34 cells were grown over poly-D-lysine-coated (0.1 mg/mL, Sigma-Aldrich, San Luis, MO, USA; ID P6407) coverslips. Prior to fixation, for SUnSET experiments, cells were treated with 1 µg/mL puromycin during 1 h. The medium was removed from wells, and following a wash with PBS pH 7.4, cells were fixed with 4% paraformaldehyde (Sigma-Aldrich, San Luis, MO, USA; 158127) for 15 min at room temperature. After PBS washes, cells were permeabilized and blocked in PBS containing 0.3% Triton X100 (Sigma-Aldrich, San Luis, MO, USA; ID X100) with 3% bovine serum albumin (NZYTech, Lisboa, Portugal; ID MB046) for 1 h. Coverslips were incubated with the suitable antibodies (Table 2), diluted in blocking solution overnight at 4 °C and washed with PBS. Coverslips were incubated with the appropriate secondary antibodies conjugated with Alexa Fluor dyes (Invitrogen, Waltham, MA, USA) for 1 h at room temperature. Nuclei were counterstained with 1 µM 4′-6-diamino-2-phenylindole (DAPI, Invitrogen, Waltham, MA, USA; ID D1306) diluted in PBS for 10 min and washed with PBS. Coverslips were mounted using ProLong^TM^ Gold Antifade Mountant (Invitrogen, Waltham, MA, USA; ID P36930) and dried for 24 h before visualization. Fluorescence was visualized using a Nikon Eclipse 90i fluorescence microscope (Nikon, Tokyo, Japan) or a Leica TCS SP5 Confocal Microscope (Leica Microsystems, Wetzlar, Germany). Images from confocal microscopy were presented as maximal projections. Image analysis was performed with Fiji software [24].

### 2.5. Superoxide Generation and Mitochondrial Membrane Potential Measurement

Intracellular and mitochondrial superoxide anion levels were assessed using dihydroethidium (HE, Invitrogen, Waltham, MA, USA; ID D11347) or MitoSOX Red (Invitrogen, Waltham, MA, USA; ID M36008), respectively. The mitochondrial membrane potential was assessed using MitoTracker Red FM (Invitrogen, Waltham, MA, USA; ID M22425). For flow cytometric analysis, cells were incubated with 2 µM HE for 60 min, 1 µM MitoSOX for 30 min or 0.5 µM MitoTracker Red FM for 30 min. Cells were washed once in PBS, detached, and centrifuged for 5 min at 250 × g. Pellets were resuspended in an appropriate volume of PBS and dead cells were stained with 1 µM DAPI or 2 µM TO-PRO-3 Iodide (Invitrogen, Waltham, MA, USA; ID T3602). Then, fluorescence was recorded in a BD FACSCanto^TM^ II Cell Analyzer (BD Biosciences, Franklin Lakes, NJ, USA). A data analysis was performed with Kaluza software (Beckman Coulter, Pasadena, CA, USA).

### 2.6. Cell Viability Assessed by MTT Reduction and Annexin-V/Propidium Iodide

The tetrazolium ring of 3-(4,5-dimethylthiazol-2-yl)-2,5-diphenyltetrazolium bromide (MTT) can be reduced with active dehydrogenases to produce a formazan precipitate. Cells were incubated in serum-free media with MTT (0.2 mg/mL) for 45 min at 37 °C. Thereafter, the media were removed and DMSO added to each well to dissolve the formazan precipitate for 20 min with gentle agitation. A total of 100 µL of the supernatants was analyzed in 96-well multiwell plates at 550 nm using a VERSAmax microplate reader (Molecular Devices, San Jose, CA, USA). For Annexin-V-FITC staining, the cells were washed twice with Annexin-V binding buffer (containing 10 mM HEPES-NaOH, pH 7.4, 150 mM NaCl, 5 mM KCl, 1 mM MgCl_2_, 1.8 mM CaCl_2_), resuspended in 100 μl of a 1:100 dilution of TACS Annexin-V-FITC (R&D Systems, Biotechne, Minneapolis, MN, USA; ID 4830-01-K) in Annexin-V binding buffer containing 1 μg/mL Propidium Iodide (PI) and incubated for 15 min at room temperature. Fluorescence was measured with a CytoFLEX flow cytometer (Beckman Coulter, Pasadena, CA, USA), by analyzing at least 10,000 events per condition. The data analysis was performed using Kaluza softwar (Beckman Coulter, Pasadena, CA, USA).

### 2.7. Cell Cycle Analysis

For EdU analysis, the Click–iT™ EdU Alexa Fluor™ 647 Flow Cytometry Assay Kit (Invitrogen, Waltham, MA, USA; ID C10424) was used following the manufacturer’s instructions. Briefly, cells were treated with 10 µM EdU for 2 h, harvested, fixed, and permeabilized. For analysis of DNA content, cells were counterstained with 1µg/mL Hoechst 33342 (Invitrogen, Waltham, MA, USA; ID H3570) for 30 min in darkness. Analysis of EdU incorporation and DNA content was performed with a CytoFLEX flow cytometer (Beckman Coulter, Pasadena, CA, USA), by analyzing at least 10,000 events per condition. The data analysis was performed using Kaluza software (Beckman Coulter, Pasadena, CA, USA).

### 2.8. Immunoblotting

Cells were washed with cold PBS and homogenized in lysis buffer (50 mM Tris pH 7.6, 400 mM NaCl, 1 mM EDTA, 1 mM EGTA and 1% SDS). Samples were heated at 95 °C for 15 min and sonicated. Protein quantification was performed with the DC^TM^ Protein Assay (Bio-Rad, Hercules, CA, USA; ID 5000112), and protein loading buffer (50 mM Tris-HCl pH 6.8, 2% SDS, 0.1% bromophenol blue, 10 % glycerol, 150 mM β-mercaptoethanol) was added. Samples were boiled at 95 °C and cellular debris was cleared with centrifugation. Proteins were resolved using SDS-PAGE and transferred to 0.45 µm pore size Immobilion-P membranes (Millipore, Burlington, MA, USA; ID IPVH00010). For immunoblotting, membranes were hydrated in methanol, washed in TTBS (20 mM Tris-HCl pH 7.5, 150 mM NaCl and 0.1% Tween 20) buffer and blocked with 5% non-fat dry milk in TTBS. Membranes were incubated with the appropriate dilution of the primary antibodies (Table 2) in 0.4% BSA or 2.5% non-fat-dry milk for 1 h, washed and incubated with 1:10,000 dilution of secondary antibodies coupled to horseradish peroxidase in 0.4% BSA TTBS for 1 h. Proteins were detected by carrying out enhanced chemiluminescence (Amersham^TM^ ECL^TM^ Select Western Blotting Detection Reagent, GE Healthcare, Chicago, IL, USA; ID GERPN2235). 

### 2.9. mRNA Quantification by qRT-PCR

Total RNA was extracted using TRI^®^ Reagent (Invitrogen, Waltham, MA, USA; ID AM9738) according to the manufacturer’s instructions. One μg of RNA (quantified using Nanodrop™ 2000 Spectrophotometer) was reverse-transcribed in a 20 μL volume using the High-Capacity RNA to cDNA^TM^ kit (Invitrogen, Waltham, MA, USA; ID 4387406). For qPCR, the reaction was performed in 10 μL using the fluorescent dye Power SYBR^TM^ Green PCR Master Mix (Applied Biosystems, Waltham, MA, USA; ID 4367659) and a mixture of 5 pmol of reverse and forward primers. Primer pairs are listed in Table 3. Quantification was performed using a StepOne detection system (Applied Biosystems, Waltham, MA, USA). PCR cycles proceeded as follows: initial denaturation for 10 min at 95 °C, then 40 cycles of denaturation (15 s, 95 °C), annealing (30 s, 60 °C) and extension (30 s, 60 °C). Data analysis is based on the ΔΔCT method with normalization of the raw data to the geometric mean of housekeeping genes. For intron analysis of the *Nfe2l2* transcript, RNA was pretreated with DNase I (Invitrogen, Waltham, MA, USA; ID 18068015) according to manufacturer’s instructions before retro-transcription to avoid genomic DNA contamination. Additionally, intron levels were normalized with total *Nfe2l2* abundance using intron-spanning primers. 

### 2.10. Luciferase Assays

Transient transfections of NSC34 cells were performed with the expression vectors for TK-Renilla and ARE-LUC. Cells were seeded on 24-well plates (100,000 cells per well), cultured for 16 h, and transfected Lipofectamine 2000 (Invitrogen, Waltham, MA, USA; ID 11668019) in OptiMEM (Gibco, Waltham, MA, USA; ID 31985062) at a ratio of 1:1 (μL lipofectamine:μg DNA) according to the manufacturer’s instructions. After 48 h of recovery from transfection, the cells were lysed and assayed with a dual-luciferase assay system (Promega, Madison, WI, USA; ID E1910) according to the manufacturer’s instructions. Relative light units were measured in a GloMax 96 microplate luminometer with dual injectors (Promega, Madison, WI, USA)

### 2.11. RNA Immunoprecipitation

Transfected NSC34 cells were detached in cold PBS and pelleted by performing centrifugation. Cells were lysed in 1 mL of RNA immunoprecipitation lysis buffer (Tris-HCl pH 7.4 25 mM, KCl 150 mM, EDTA 0.5 mM, NP40 0.5%, RNAseOUT 100 U/mL (Invitrogen, Waltham, MA, USA; ID 10777019), 1 mM phenylmethylsulfonyl fluoride and 1 µg/mL leupeptin (Sigma-Aldrich, San Luis, MO, USA; IDs P7626 and L5793) at 4 °C for 30 min with end-over-end rotation. Lysates were cleared by centrifugation at 13,000× *g* for 15 min and supernatants were collected. Lysates were divided in half for immunoprecipitation with 5 µg of anti-GFP antibody or with 5 µg of IgG isotype control overnight at 4 °C with end-over-end rotation. Immunocomplexes were isolated using Protein G Sepharose^®^ 4 Fast Flow (Cytiva, Danaher Corporation, Washington DC, WA, USA; ID GE17-0618-01) during 1 h and beads with immunocomplexes washed three times with 500 µL of RIP lysis buffer. Before elution, a sample was taken to ensure EGFP immunoprecipitation with Western blot. For elution of RNA from immunocomplexes, TRI^®^ Reagent was used according to the manufacturer’s instructions. RNA isolates were quantified and treated with appropriate amounts of DNAse I according to the manufacturer’s instructions. Retrotranscription and qPCR analysis were performed as described above.

### 2.12. Statistical Analyses

Unless otherwise indicated, all experiments were performed at least 3 times and all data presented in the graphs are the mean of at least 3 independent samples. Data are presented as mean ± SEM (standard error of the mean). Statistical differences between groups were assessed using GraphPad Prism 8 by one and two-way analyses of variance, using Dunnett’s post-hoc test when comparing transfections/peptide treatments to EGFP or HA controls and Bonferroni’s post-hoc test when comparing transductions/stable cell lines to Sham or Control transduction (**** indicates *p* values <0.0001, ***, *p* < 0.001, ** *p* < 0.01 and * *p* < 0.05).

## 3. Results

### 3.1. C9orf72 Patient Leukocytes Exhibit Dysfunctional Expression of Several Redox Genes

**Figure 1:**
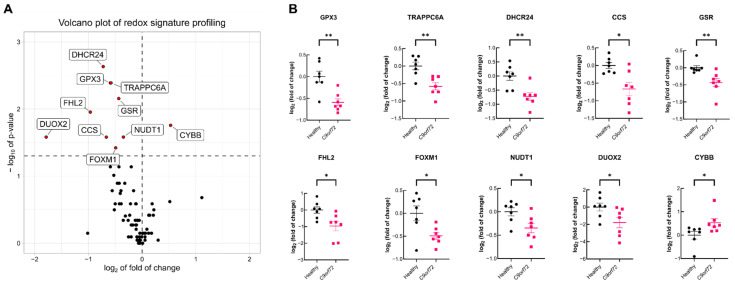
Expression of redox signature is impaired in leukocytes from *C9orf72* patients. mRNA levels for redox genes were determined with qRT-PCR in leukocyte RNA from healthy donors (*n* = 7) or *C9orf72* ALS patients (*n* = 7) and normalized using the geometric mean of the levels of the ACTB, B2M, GAPDH, HPRT1 and RPLP0 as housekeeping genes. (**A**) Volcano plot depicting the mean log_2_ of the fold of change of each tested gene and −log_10_ *p*-value between *C9orf72* and healthy individuals. Horizontal dashed line indicates *p* < 0.05, and vertical dashed line indicates log_2_ fold of change = 0. (**B**) log_2_ fold of change of the expression of the genes with statistically significant changes in each individual. Horizontal bars indicate the mean log_2_ fold of change value for the mRNA levels in each group and error bars indicate the SEM. Individual points indicate the expression of that gene in each individual. Statistical analysis was performed with the Wilcoxon rank-sum non-parametric test. * *p* < 0.05; **, *p* < 0.01 comparing ALS vs. control group.

Blood provides a window for investigating the inter-related systemic and local immune responses, inflammation and redox signaling alterations in chronic pathologies [25,26]. We investigated whether alterations in redox-related genes could be measurable in peripheral blood from *C9orf72*-ALS patients, as a reflection of gene expression changes occurring in the brain and spinal cord. We conducted a case-control study on seven *C9orf72*-ALS patients and seven healthy controls who were highly characterized and matched in terms of age and gender (Table 1). Targeted gene expression screening was performed with qRT-PCR using a pathway-focused array of 84 redox genes on leukocyte RNA (Supplementary Table S2). As shown in the gene expression volcano plot (Figure 1A), we identified nine redox genes that were significantly downregulated and one gene that was upregulated (Wilcoxon rank-sum test, *p* < 0.05) in the leukocytes of patients vs. controls. These genes encode members of the NADPH oxidases family (CYBB and DUOX2) that are involved in ROS production (Figure 1B), proteins that regulate the metabolism of glutathione (GSR and GPX3), or perform other redox functions (DHCR24, FHL2, CCS, FOXM1, TRAPPC6A, NUDT1). These results suggest a global impairment of redox homeostasis in the blood of *C9orf72*-ALS patients.

### 3.2. Arg-DPRs Lead to Decreased Mitochondrial Membrane Potential and Cell Death

**Figure 2:**
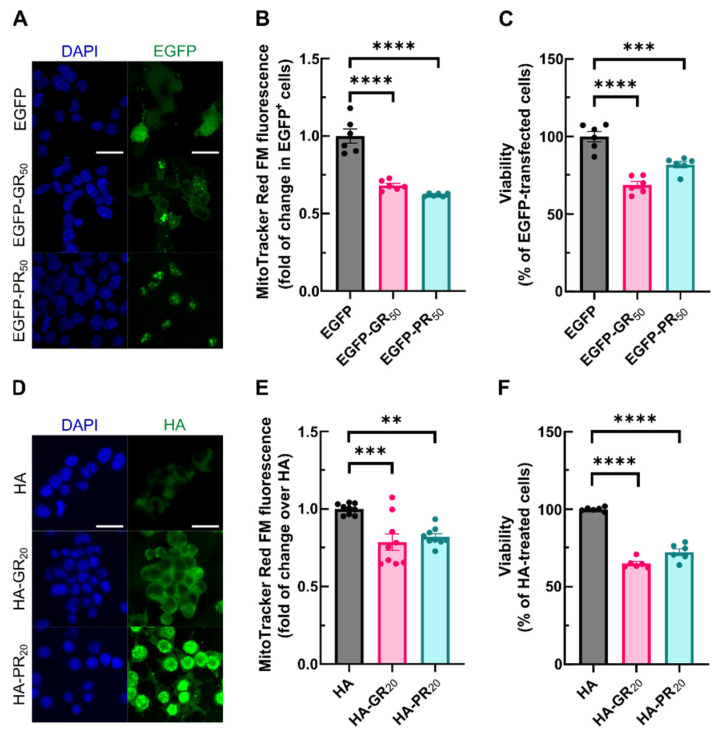
Endogenous and exogenous exposure to poly-GR and poly-PR decrease mitochondrial membrane potential and induce cell death. NSC34 cells were either transiently transfected with EGFP (control), EGFP-GR_50_ or EGFP-PR_50_ during 48 h or treated with 6 µM of HA, HA-GR_20_ or HA-PR_20_ peptides for 6 h (immunofluorescence) or 24 h (flow cytometry and viability assay). (**A**,**D**) EGFP fluorescence or HA immunofluorescence staining was analyzed by microscopy. Nuclei were counterstained with 4′,6-diamidino-2-phenylindole (DAPI). Scale bar: 40 µm. (**B**,**E**) cells were treated with 0.5 µM MitoTracker Red FM during 30 min and fluorescence was measured by flow cytometry analysis. Each point depicts the geometric mean of probe fluorescence in cells from that sample ((**B**), alive EGFP^+^ cells, (**E**), all alive cells). (**C**,**F**) viability of the cells was measured using an MTT assay. Bar height indicates the mean of the whole group and error bars indicate the SEM. Statistical analysis was performed with one-way ANOVA. Dunnett’s post-hoc test. **, *p* < 0.01; ***, *p* < 0.001; ****, *p* < 0.0001.

The hallmark of *C9orf72*-related pathology is the transcription of hexanucleotide expansions that produce toxic dipeptide repeat proteins. In a reverse translational approach, we aimed to correlate the redox alterations in *C9orf72*-ALS patients with this pathological mechanism in a simplified model of DPR expression using cultured the NSC34 motor neuronal cell line. NSC34 cells were transfected with expression vectors for either enhanced green fluorescent protein (EGFP), or EGFP fused to a 50-long dipeptide repeat of poly-GR (EGFP-GR_50_) or poly-PR (EGFP-PR_50_). EGFP immunofluorescence was analyzed 48 h following transfection. EGFP exhibited a diffuse pattern of expression in both cytosol and nuclei, whereas EGFP-GR_50_ and EGFP-PR_50_ were preferentially accumulated in nuclear dots (Figure 2A), mimicking the characteristic accumulation in nucleolar structures of DRPs [27]. Since mitochondria are a central organelle for redox biology, first, we attempted to address the impact of Arg-DPRs on mitochondrial membrane potential (ΔΨm). A flow cytometry evaluation of ΔΨm with MitoTracker Red FM revealed that the overexpression of EGFP-GR_50_ or EGFP-PR_50_ reduced the mitochondrial potential by 30% compared to EGFP-cells (Figure 2B). As an alternative approach, and by taking advantage of the cell-penetrating properties of arginine-containing peptides [28], we conducted similar experiments in this cell line submitted to 6 μM of either HA control peptide, HA-tagged 20-long dipeptide repeat poly-GR (HA-GR_20_) or poly-PR (HA-PR_20_). Immunodetection with anti-HA antibodies showed puncta accumulation of HA-GR_20_ and HA-PR_20_ in agreement with previous reports [29,30] (Figure 2D). When NSC34 cells were exposed to HA-GR_20_ or HA-PR_20_ peptides during 24 h, a 20% drop in MitoTracker Red FM fluorescence was observed in HA-GR_20_ or HA-PR_20_ compared to HA-treated cells (Figure 2E). Since a long-lasting drop in ΔΨm may induce cell death, we evaluated cell viability with an MTT assay. A reduction in the formazan signal, measured with the MTT assay, may derive from an increase in cell death but also an inhibition in proliferation. For this reason, we validated that the Arg-DPRs did not modify NSC34 cell cycle progression by analyzing EdU incorporation and DNA content. Although cell cycle was not altered in any condition, there was an increase in the percentage of cell death upon Arg-DPRs exposure, which we found by analyzing Annexin-V-FITC and Propidium Iodide (IP) staining (Supplementary Materials; Figure S1). As shown in Figure 2C, the overexpression of EGFP-GR_50_ and EGFP-PR_50_ reduced the survival rate by 40% compared with EGFP-transfected cells. Accordingly, HA-GR_20_ and HA-PR_20_ reduced the survival rate to the same extent compared with HA-treated cells (Figure 2F). Taken together, these findings link cellular death with a decrease/reduction in the mitochondrial membrane potential induced by Arg-DPRs.

### 3.3. Arg-Containing Dipeptide Repeats from the C9orf72 Expansion Disrupt Redox Homeostasis

**Figure 3:**
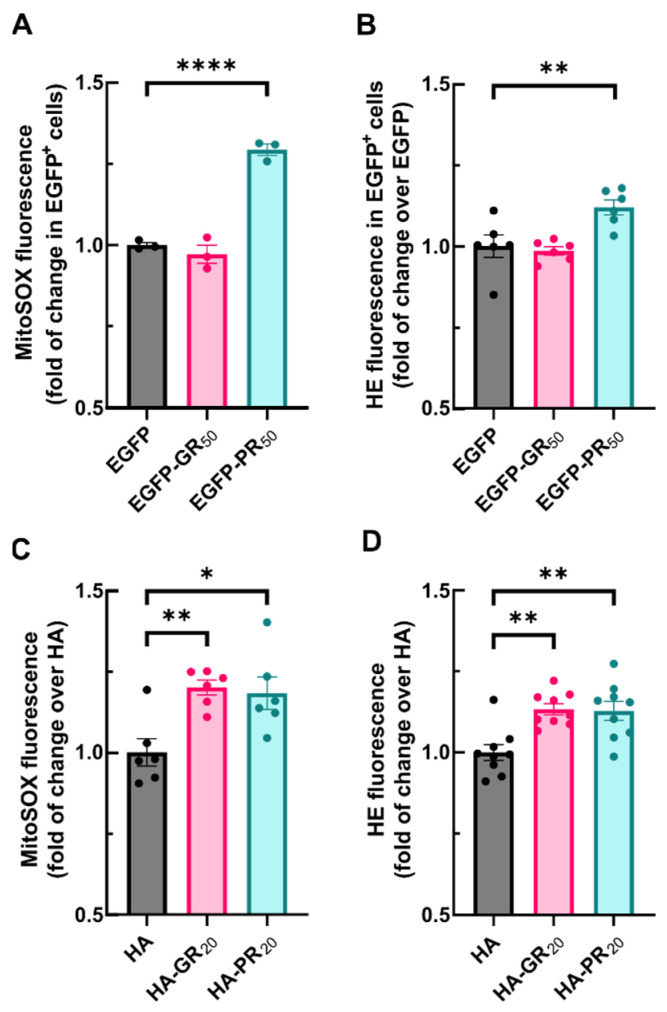
DPRs exposure leads to mitochondrial and intracellular superoxide generation in NSC34 cells. (**A**) NSC34 cells were transiently transfected with EGFP (control), EGFP-GR_50_ or EGFP-PR_50_ during 48 h. or treated with 6 µM of HA, HA-GR_20_ or HA-PR_20_ peptides for 2 h. (**A**,**C**) Cells were treated with 1 µM MitoSOX during 30 min and fluorescence was measured by flow cytometry analysis. (**B**,**D**) cells were treated with 2 µM hydroethidine during the last hour of transfection/treatment and probe intensity was measured by flow cytometry analysis. Each point depicts the geometric mean of probe fluorescence in cells from that sample ((**A**,**B**), alive/EGFP^+^ cells, (**C**,**D**), all alive cells). Bar height indicates the mean of the whole group and error bars indicate the SEM. Statistical analysis was performed with one-way ANOVA. Dunnett’s post-hoc test. *, *p* < 0.05; **, *p* < 0.01; ****, *p* < 0.0001.

The dysregulation of mitochondrial membrane potential is closely related to the maintenance of high levels of reactive oxygen species (ROS). Therefore, we next analyzed the levels of superoxide ions inside mitochondria with MitoSOX Red. NSC34 was transfected with either EGFP, EGFP-GR_50_ or EGFP-PR_50_. The flow cytometry analysis revealed that, although the overexpression of EGFP-GR_50_ did not modify superoxide levels, overexpression of EGFP-PR_50_ led to a slight but significant increase in superoxide levels (Figure 3A). Then, we evaluated the effect of exogenous Arg-DPRs peptides on mitochondrial superoxide levels. NSC34 cells were submitted to either HA, HA-GR_20_ or HA-PR_20_ over 2 h and stained with MitoSOX Red. In this experimental setting, mitochondrial superoxide levels were higher in both HA-GR_20_ and HA-PR_20_ compared with HA-treated cells (Figure 3C). Accordingly, the overexpression of EGFP-DPRs (Figure 3B) or treatment with exogenous Arg-DPRs peptides (Figure 3D) led to a similar rise in the fluorescence derived from the oxidation of another redox sensitive probe, hydroethidine (HE). These results provide strong evidence that Arg-DPRs increase ROS levels in NSC34 motor neurons, probably contributing to mitochondrial damage. 

### 3.4. Activation of NRF2 Is Impeded by DPRs

**Figure 4:**
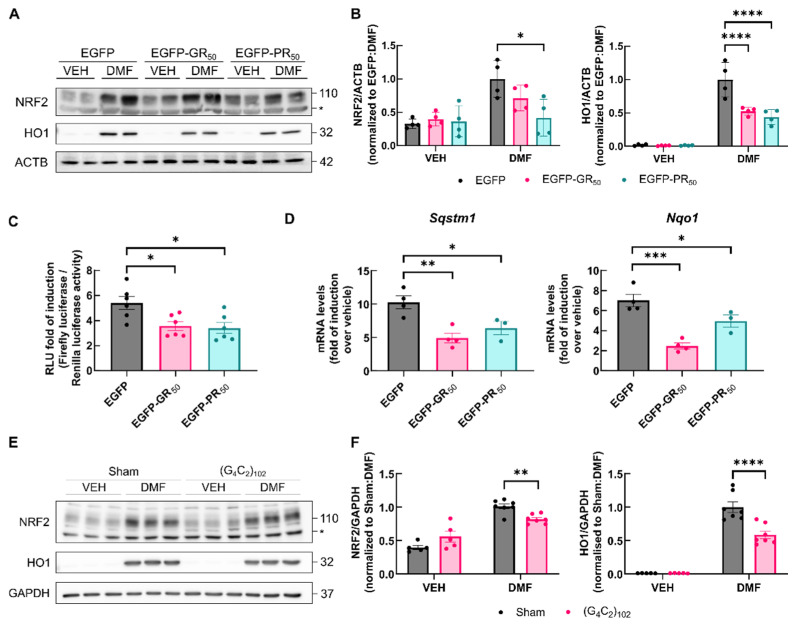
Pharmacological activation of NRF2-dependent transcription by dimethyl fumarate is impaired in EGFP-GR_50_, EGFP-PR_50_ or (G_4_C_2_)_102_ expressing cells. (**A**) NSC34 cells were transiently transfected with EGFP, EGFP-GR_50_ or EGFP-PR_50_ for 48 h and treated with either VEH (DMSO 0.1%) or DMF 100 µM over the last 16 h. (**A**) The levels of the indicated proteins were measured by performing an immunoblot analysis. ACTB levels were determined as a loading control. (**B**) Densitometric quantification for the immunoblots for NRF2 and HO1 presented in (**A**). (**C**) NSC34 cells were transiently co-transfected with EGFP, EGFP-GR_50_ and EGFP-PR_50_ and plasmids encoding for ARE-luciferase reporter and TK-Renilla luciferase for 48 h and treated as before. Fold of induction over vehicle in relative luciferase units is shown. (**D**) Fold of induction on mRNA levels of the *Sqstm1* and *Nqo1* genes in EGFP-DPRs transfected cells treated with DMF. mRNA levels were normalized using the geometric mean of the housekeeping genes *Gapdh* and *Actb* and to the non-induced control. (**E**) The expression of the sham cassette or the (G_4_C_2_)_102_ cassette was induced for 7 days with 1 µg/mL tetracycline in NSC34 cells. In the last 16 h, cells were treated with either VEH (DMSO 0.1%) or DMF 100 µM. The levels of the indicated proteins were measured by performing an immunoblot analysis. GAPDH levels were determined as a loading control. (**F**) Densitometric quantification for the immunoblots for NRF2 and HO1 presented in (**C**). Individual points in (**B**,**D**) represent the protein levels normalized by protein amount in each lane, bar height represents the mean of those protein levels in each group and error bars represent the SEM. Statistical analysis was performed with two-way ANOVA. Dunnett’s post-hoc test in (**B**–**D**). Bonferroni’s post-hoc test in (**F**). *, *p* < 0.05; **, *p* < 0.01; ***, *p* < 0.001; ****, *p* < 0.0001.

Next, we investigated the potential impact of Arg-DPRs on NRF2 activation. NSC34 cells were transfected with either EGFP, EGFP-GR_50_ or EGFP-PR_50_; after 48 h, cells were treated with dimethyl fumarate (DMF; 100 μM DMF for 16 h) to inhibit KEAP1. As expected, DMF led to a robust increase in both NRF2 and HO-1 in EGFP-cells. Interestingly, this increase was reduced in EGFP-GR_50_ or EGFP-PR_50_ cells compared to EGFP cells (Figure 4A,B). The increase in the NRF2 protein levels correlated with higher transactivating activity as determined with an ARE-driven luciferase-reporter construct, 3xARE-LUC, created from the NRF2 responsive ARE in the mouse *Hmox1* promoter [31]. NSC34 cells were co-transfected with EGFP, EGFP-GR_50_ or EGFP-PR_50_ together with ARE-firefly luciferase and TK-Renilla reporters. After overnight recovery, they were stimulated for 16 h with DMF. As shown in Figure 4C, DMF increased reporter gene activity by about 5-fold in EGFP control cells. By contrast, the overexpression of either EGFP-GR_50_ or EGFP-PR_50_ yielded a reduced 3-fold increase. Concordantly, the induction of two verified NRF2 targets, *Sqstm1* and *Nqo1*, exhibited reduced transcript levels upon DMF treatment in EGFP-GR_50_ and EGFP-PR_50_ cells compared to EGFP cells (Figure 4D). As an additional confirmation of our results, we analyzed NRF2 activation in a chronic model of DPR exposure based on a tetracycline-inducible cassette that contains 102 repeats of the (G_4_C_2_) hexanucleotide and produces only the sense strand of the toxic RNA species [32]. To determine the status of NRF2/HO-1, sham and (G_4_C_2_)_102_ cells were treated with tetracycline for 7 days, and then submitted to 100 μM DMF for 16 h. NRF2 and HO-1 protein levels were measured in response to NRF2 activation. DMF prompted a robust increase in both NRF2 and HO-1 protein levels; however, this induction was significantly decreased in (G_4_C_2_)_102_ bearing cells (Figure 4E,F). Taken together, these results point to an impairment in NRF2 activation induced by Arg-DPRs.

### 3.5. Accumulation of NRF2 Transcription Factor Is Impaired by DPRs

**Figure 5:**
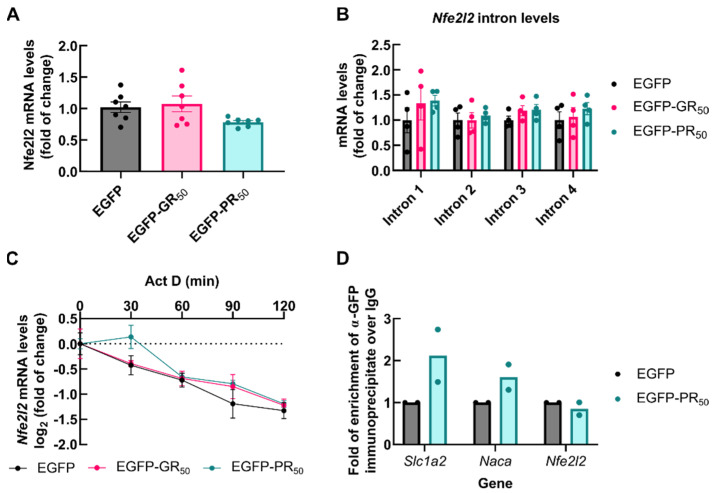
DPRs do not modify *Nfe2l2* mRNA levels, splicing, half-life or sequester them. NSC34 cells were transiently transfected with EGFP (control), EGFP-GR_50_ or EGFP-PR_50_ for 48h. (**A**,**B**) *Nfe2l2* total transcripts levels and intron levels in mRNA were measured by qRT-PCR. (**C**) Transcription was blocked with 5 µg/mL of actinomycin D in transfected NSC34 cells for the indicated times and mRNA levels for *Nfe2l2* were measured by qRT-PCR. (**D**) mRNA levels of the indicated transcripts were measured by qRT-PCR in RNA bound to EGFP-immunocomplexes. In (**A**,**B**) mRNA levels were normalized by the geometric mean of the housekeeping genes *Gapdh*, *Actb* and *Tbp.* In (**C**) mRNA levels were normalized by the geometric mean of the stable transcripts from *Gapdh* and *Actb*. Bar height (**A**,**B**,**D**) and point (**C**) indicates the mean of the whole group and error bars indicate the SEM. Statistical analysis was performed with one-way ANOVA for (**A**,**B**), and two-way ANOVA in (**C**), yielding non-significant changes.

Since the transactivation of ARE-containing genes with DMF depends on newly synthetized NRF2 [33], we examined the transcription, splicing and stability of *Nfe2l2* mRNA. NSC34 cells were transiently transfected with EGFP, EGFP-GR_50_ or EGFP-PR_50_. At 48 h following transfection, no change was found in immature or mature mRNA levels of *Nfe2l2* when comparing samples from EGFP-GR_50_ and EGFP-PR_50_ to EGFP cells (Figure 5A,B). The degradation rate of *Nfe2l2* mRNA was also analyzed using an actinomycin D chase assay. Gene transcription was blocked with 5 µg/mL actinomycin D and the levels of *Nfe2l2* were measured with qRT-PCR. As shown in Figure 5C, *Nfe2l2* mRNA exhibited a half-life of 85 min in EGFP [34], which was not altered by the overexpression of EGFP-GR_50_ or EGFP-PR_50_. We also analyzed whether the Arg-DPRs nuclear aggregates might sequester the *Nfe2l2* transcripts. We performed RNA immunoprecipitation by employing anti-GFP or control IgG antibody, and the levels of *Nfe2l2* mRNA bound in the immunocomplexes were determined with qRT-PCR. As shown in Figure 5D, *Slc1a2* and *Naca* transcripts, used as controls [29], were enriched in EGFP-PR_50_ immunoprecipitates compared to the EGFP. In contrast, *Nfe2l2* mRNA was not enriched in the same complexes.

**Figure 6:**
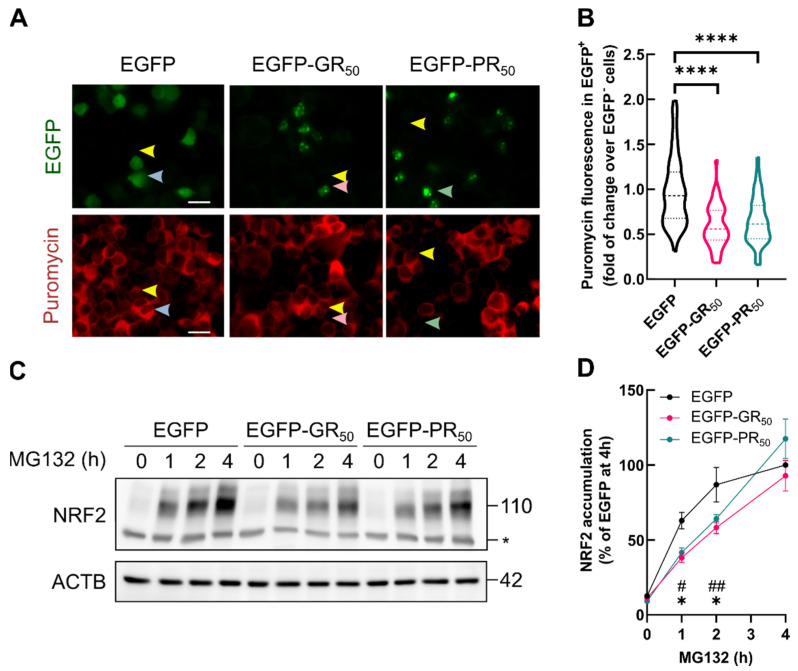
General translational shutdown caused by DPRs impairs NRF2 induction by MG132. NSC34 cells were transiently transfected with EGFP, EGFP-GR_50_ or EGFP-PR_50_. (**A**,**B**) transfected NSC34 cells were treated with 1 µg/mL puromycin for 1 h. (**A**) immunocytochemical detection of puromycin-labelled peptides. Yellow arrows, EGFP^−^ cells; blue arrows, EGFP^+^ cells; red arrows, EGFP-GR50^+^ cells; green arrows, EGFP-PR_50_^+^ cells. Scale-bar: 40 µm; (B) violin plots depicting the puromycin intensity distribution in at least 100 cells in EGFP, EGFP-GR_50_ and EGFP-PR_50_ conditions. (**C**) transfected cells were treated with MG132 (20 µM) for the indicated times. NRF2 levels were measured by immunoblot. ACTB was measured as a loading control. *, non-specific band. (**D**) densitometric quantification of the immunoblot presented in A. Values are expressed as % NRF2 accumulation at time 4 h. In (B) dotted lines represent the median and the 1st and 3rd quartile. Statistical analysis was performed with one-way ANOVA. Dunnett’s post-hoc test. ****, *p* < 0.0001. In (**D**) the point represents the mean and error bars represent SEM. Statistical analysis was performed with two-way ANOVA. Dunnett’s post-hoc test. #, EGFP vs. EGFP-GR_50;_ *, EGFP vs. EGFP-PR_50._ * or #, *p* < 0.05; ##, *p* < 0.01.

Then, we used a SUnSET assay [35] to evaluate protein translation in EGFP, EGFP-GR_50_ or EGFP-PR_50_ expressing cells. Transfected cells were treated with 1 µg/mL puromycin for 1 h. During this period, puromycin is incorporated into the C-terminus of nascent proteins; then, its levels can be detected by immunocytochemistry. As shown in Figure 6A,B, puromycin fluorescence was diminished in EGFP-GR_50_ and EGFP-PR_50_ expressing cells compared to EGFP. These data are in line with previous reports [29,36], supporting the fact that Arg-DPRs lead to a decrease in general protein synthesis. Then, we blocked NRF2 proteasomal degradation to specifically determine the impact of Arg-DPRs on NRF2 protein synthesis. NSC34 transfected with EGFP, EGFP-GR_50_ or EGFP-PR_50_ were submitted to MG132 at the indicated times. As shown in Figure 6C,D, NRF2 levels increased as expected in EGFP-overexpressing cells; importantly, the expression of EGFP-GR_50_ or EGFP-PR_50_ resulted in significantly smaller NRF2 accumulation. Taken together, these data indicate that the translational impairment induced by Arg-DPRs directly impacts NRF2 synthesis, resulting in reduced transcriptional activity.

### 3.6. NRF2 Activation Protects against Arg-DPR Toxicity in NSC34 Cells

**Figure 7:**
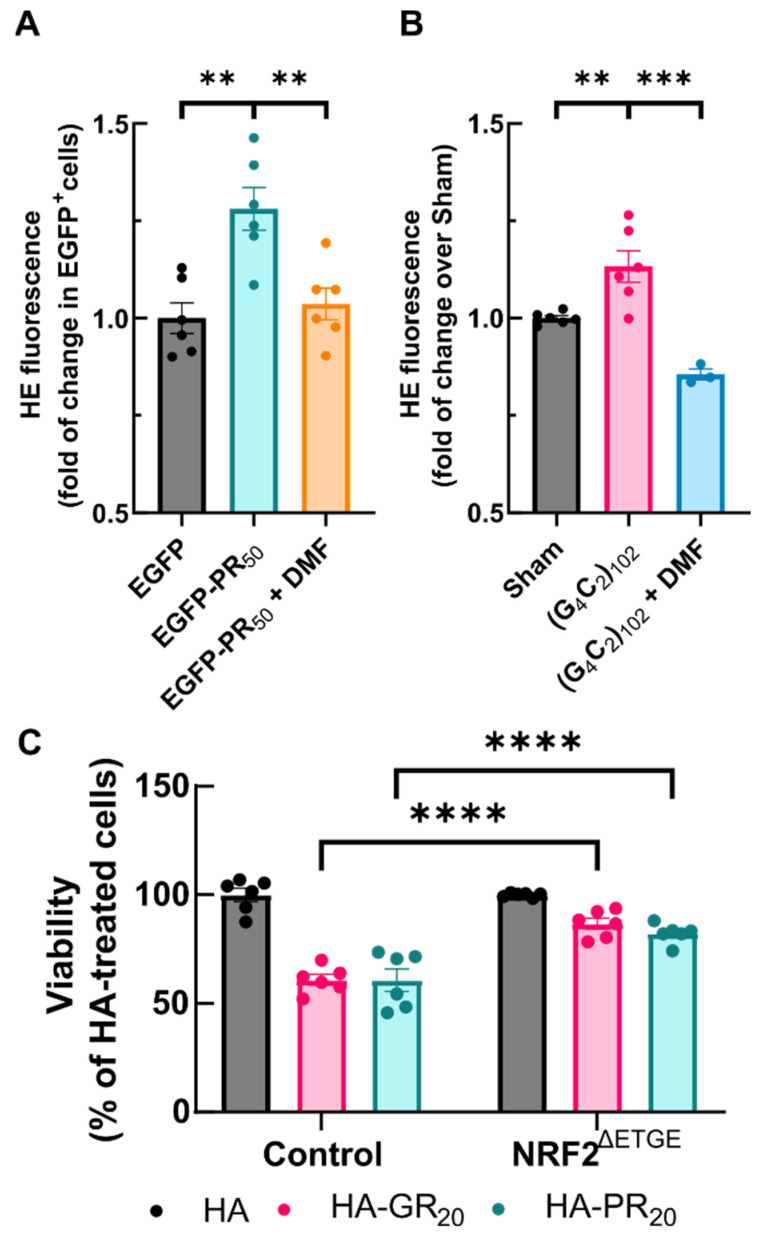
Activation of NRF2 rescues from DPRs toxicity. (**A**,**B**) NSC34 cells were transiently transfected with EGFP, EGFP-GR_50_ or EGFP-PR_50_ for 48 h or the expression of the Sham cassette or the (G_4_C_2_)_102_ cassette was induced for 7 days with 1 µg/mL tetracycline in NSC34 cells, respectively. Cells were treated with either VEH (DMSO 0.1%) or DMF 100 µM over the last 4 h and stained with 2 µM hydroethidine during the last hour. Probe intensity was measured by flow cytometry analysis. In (**A**,**B**), each point depicts the geometric mean of probe fluorescence in cells from that sample (**A**), alive EGFP^+^ cells, (**B**), all alive cells). (**C**) NSC34 cells were transduced with either control lentiviral particles or encoding the stable NRF2 version lacking the ETGE KEAP1-binding motif for 48 h and then treated with 6 µM of HA, HA-GR_20_ or HA-PR_20_ peptides for 24 h. Cell viability was measured using a MTT assay Bar height indicates the mean of the whole group and error bars indicate the SEM. Statistical analysis was performed with one-way ANOVA (**A**,**B**), Dunnett’s post-hoc test or two-way ANOVA (**C**), Bonferroni’s post-hoc test. ** *p* < 0.01; ***, *p* < 0.001; ****, *p* < 0.0001.

Despite the delay in NRF2 activation caused by DPRs, we investigated whether the NRF2 system was responsive enough to counteract ROS induction by Arg-DPRs. To test this hypothesis, NSC34 transfected with EGFP and EGFP-PR_50_ were submitted to 100 µM DMF for 4 h and HE fluorescence was assessed with flow cytometry. As shown in Figure 7A, DMF treatment abrogated the increase in HE fluorescence produced by EGFP-PR_50_ expression. We replicated these results in our stably transfected NSC34 (G_4_C_2_)_102_ model induced for 7 days (Figure 7B). NSC34 (G_4_C_2_)_102_ showed a slight but significant increase in superoxide levels compared to sham cells, that were rescued upon DMF treatment for 4 h.

Next, we questioned whether supplementation of NRF2 levels with the overexpression of a stable version of NRF2 (NRF2^ΔETGE^) might improve the survival rate of NSC34 cells submitted to the Arg-DPR challenge. NSC34 cells were infected with either control lentiviral vectors for NRF2^ΔETGE^ for 24 h, and then treated with HA, HA-GR_20_ or HA-PR_20_ peptides for an additional 24 h. As shown in Figure 7C, Arg-DPRs peptides led to a 40% reduction in NSC34 viability, measured with the MTT assay, that was fully recovered by overexpressing NRF2 (Supplementary Figure S2). These results provide a proof of concept indicating that NRF2 activation could have therapeutic potential in *C9orf72*-related ALS by protecting motor neurons from Arg-DPR toxicity.

## 4. Discussion

In the present study, we investigated the redox, mitochondrial and NRF2 status in the context of dipeptide repeat protein toxicity resulting from pathological hexanucleotide expansion in the *C9orf72* gene. First, we performed redox signature profiling of leukocyte RNA from *C9orf72* patients, and found a dysregulation in genes involved with defense against oxidative damage compared to healthy volunteers. Secondly, we found that Arg-DPRs present in *C9orf72* patients trigger an increase in ROS at the mitochondrial level, this being coupled with a reduction in mitochondrial membrane potential in the NSC34 cell line. Interestingly, the levels of *FoxM1* and *Trappa6c* mRNA were reduced in C9orf72 patients’ leukocytes and in Arg-DPRs mouse NSC34 (data not shown). Thirdly, we described an impairment in the activation of the transcription factor NRF2 in the presence of Arg-DPRs. Finally, we describe how activation of the NRF2 transcriptional response diminishes Arg-DPR toxicity, suggesting that the activation of NRF2 in *C9orf72* may represent a potential therapeutic target.

In this study, we reported the dysregulation of ten key transcripts related to redox biology in leukocytes from *C9orf72* ALS patients compared to healthy controls for first the time. The dysregulated mRNAs could be grouped into those coding for antioxidant enzymes including GSR and peroxidases (GPX3 and DUOX2) and proteins related to superoxide metabolism (CCS and CYBB). The downregulation of CSS, a chaperone that delivers Cu2+ ions to the SOD1 protein, and up-regulation of CYBB, a membrane-bound subunit of NADPH oxidase, could potentially contribute to superoxide accumulation [37]. Hydrogen peroxide or hydroperoxides are reduced by GPX3 activity, leading to the production of oxidized glutathione (GSSG), which in turn is recycled into GSH by GSR. Adequate expression levels of GPX3 and GSR are crucial to maintain antioxidant defense and the glutathione pool and, therefore, to protect cells from oxidative damage. Moreover, we found a diminished expression of three transcripts encoded by genes whose expression is activated by ROS (DHCR24, FOXM1 and NUDT1) and two transcripts related to ROS-activated pathways (FHL2 and TRAPPC6A). Taken together, these data indicate that the antioxidant response of *C9orf72* patients is impaired compared with control subjects. These data are in agreement with previous reports which described redox dysregulation in ALS. Recently, a meta-analysis which included 41 studies with 4588 ALS patients and 6344 control individuals analyzing 15 oxidative stress markers from the blood suggests that MDA (lipid peroxidation end product), 8-OHdG (a marker for DNA damage), and AOPP (Advanced Oxidation Protein Product) were significantly elevated in the blood of ALS patients when compared with control individuals. On the contrary, this study reveals that the levels of antioxidant glutathione and uric acid were significantly downregulated in patients with ALS [38]. Future research will address whether this impairment is also measurable in leukocytes from patients with sporadic ALS or other types of familial ALS.

Although Arg-DPRs can lead to reduced mitochondrial membrane potential through different routes, the connection of mitochondrial damage, dysregulated ROS and poly-repeat dipeptides is supported by other studies. In fact, zebrafish embryos injected with poly-GR exhibited enhanced levels of mitochondrial superoxide generation [39]. ROS levels were also increased in *C9orf72*-derived neurons in an age-dependent manner. Pharmacological or genetic reduction in oxidative stress partially rescued DNA damage in *C9orf72* neurons and control neurons expressing GR_80_ [40]. Samples from the frontal cortex of mice expressing GR_80_ showed decreased activities of mitochondrial electron chain complexes I and V [41]. Closer to human pathology, higher levels of ROS and susceptibility to oxidative damage have been found in iPSC-derived astrocytes from patients [42] and skeletal myocytes [43], respectively.

The dysfunction in the oxidative stress response prompted us to analyze the response of the transcription factor NRF2, known to regulate several antioxidant enzymes and pathways related to redox metabolism. NRF2 dysfunction has already been found to be present in different ALS settings [44,45,46,47]. However, the impact of poly-dipeptide repeat proteins has not been addressed so far. In this regard, the activation of NRF2 and its transcriptional targets by DMF was partially impaired by poly-dipeptide overexpression. This fact directly connects the imbalanced ROS levels with an impaired antioxidant response in the context of Arg-DPRs. In this study, we provide evidence that HO-1 expression is not sufficiently induced in the presence of poly-repeat dipeptides, connecting for the first time HO-1 and *C9orf72* pathology. HO-1 degrades prooxidant heme into equimolar quantities of carbon monoxide, biliverdin, and iron. Biliverdin is immediately converted into bilirubin by biliverdin reductase, while iron is oxidized and sequestered by ferritin. Despite the well-known antioxidant properties of bilirubin, a novel role for HO-1 has recently been proposed of protection against oxidative injury by regulating mitochondrial quality control [48]. The expression of other NRF2-target genes, such as glyoxylase 1, has been shown to be reduced in neurons and astrocytes derived from fibroblasts of *C9orf72*-patients [49]. This correlation could indicate that the activation of NRF2 is hampered by Arg-DPRs in ALS patients, helping to explain the results recently disclosed from a clinical trial aiming to evaluate the safety and efficacy of DMF in ALS [50]. In this study, there was no significant improvement in the primary endpoint (Amyotrophic Lateral Sclerosis Functional Rating Scale-Revised), although there was a mild improvement in the patient’s neurophysiological index. In this case, a question arises. Would NRF2 dysfunction in ALS be sufficient to hinder the NRF2 cytoprotective response? We already demonstrated some protection from oxidative stress using DMF in the present study. Given that the impairment of NRF2 by Arg-DPRs is only partial, activation of the NRF2 pathway through more potent inducers could effectively protect against Arg-DPRs toxicity. Importantly, robust NRF2 overexpression protected NSC34 cells from the toxicity induced by Arg-DPRs. Therefore, a window for cytoprotection upon NRF2 stimulation may still be achievable, but further in vivo studies are needed.

Taking into consideration that free newly produced NRF2 is the main functional NRF2 species, synthesis defects likely contribute to its impaired activation, and even more so considering its short half-life. Indeed, brusatol, a drug used as a classical NRF2 inhibitor, exerts its effects through global protein synthesis inhibition [51]. In this study, we provide evidence in a motor neuron cell line that poly-dipeptide repeats downregulate global translation in agreement with previous reports employing HeLa cells or Drosophila neurons [52,53]. In basal conditions, the balance between synthesis and degradation defines the levels of the NRF2 protein. Upon proteasomal inhibition, NRF2 accumulation occurs to a lesser extent when Arg-DPRs are overexpressed, suggesting a significant impact of general translational shutdown upon NRF2 activation.

## 5. Conclusions

In this study, we characterized redox and mitochondrial disturbances in *C9orf72*-ALS together with impairment of the activation of NRF2. We propose that the general translation shutdown induced by Arg-DPRs reduces NRF2 synthesis, linking general cellular events in *C9orf72*-related ALS with specific molecular consequences. This work serves to underline the importance of NRF2 in *C9orf72*-related ALS and as a proof of concept to emphasize the importance of its activation as a potential therapeutic target, since Arg-DPRs toxicity can be prevented by NRF2 overexpression.

## Figures and Tables

**Table 1:** Human samples information.

	Age (Years)	Gender	Site of Onset	RIN ^1^ Value	FVC ^2^ (% Predicted)	ALSFRS-R ^3^	Progression Rate	Riluzole
Healthy controls	61	Male	-	9.6	-	-	-	-
	56	Male	-	9.5	-	-	-	-
	68	Male	-	9	-	-	-	-
	71	Female	-	8.6	-	-	-	-
	69	Female	-	8.4	-	-	-	-
	57	Female	-	8	-	-	-	-
	63	Female	-	7.3	-	-	-	-
**Mean ± SD**	63.6 ± 5.9	-	-	8.6 ± 0.8	-	-	-	-
*C9orf72* patients	53	Male	Bulbar	9.4	117	38	3.3	no
	73	Female	Bulbar	9.1	51	32	4.0	no
	65	Female	Limb	9	110	41	0.3	no
	67	Female	Limb	8.9	93	42	1.2	no
	58	Female	Limb	8.7	99	n/a	n/a	yes
	61	Male	Limb	8.5	92	41	1.0	no
	70	Female	Limb	8	70	40	0.4	no
**Mean ± SD**	63.9 ± 7.0	-	-	8.8 ± 0.5	90.3 ± 22.9	39.0 ± 3.7	1.7 ± 1.6	-

^1^ RIN, RNA integrity number; ^2^ FVC, forced vital capacity; ^3^ ALSFSR-R; revised Amyotrophic Lateral Sclerosis Functional Rating Scale.

**Table 2:** Antibodies used throughout the study.

Epitope	Dilution	Species	Source	Reference
NRF2	1:5000	Rabbit	Homemade	-
HO1	1:2000	Rabbit	Homemade	-
GFP	1:1000	Mouse	Sigma Aldrich	G1546
ACTB	1:4000	Goat	Santa Cruz Biotech.	sc-1616
GAPDH	1:30000	Mouse	Calbiochem	CB1001
HA	1:500	Mouse	Abcam	ab9110
Puromycin	1:1000	Mouse	Sigma Aldrich	MABE343

**Table 3:** qPCR primers used throughout the study.

	Gene	Forward Primer (5′—3′)	Reverse Primer (5′—3′)
*Nfe2l2*	Mature	CCCGAAGCACGCTGAAGGCA	CCAGGCGGTGGGTCTCCGTA
	Intron 1	TTTATGACCAAATACCGAGCACA	GGCTCAATGTCTGGTAACATCC
	Intron 2	AGCACAGGGTCACAACGAG	CGCTGTTCGTTAAAGGGGAG
	Intron 3	CCAAGGCATGAAGAACAGACA	TAATATGGAGTCACTGGGAGGA
	Intron 4	TGTGTGTTAAGTGAGTCTGGG	TAGGAGTGTGCTGTTTTCTGC
*Actb*		CACCCAGCACATTTAGCTAGCTGA	TTCAGAGCAACTGCCCTGAAAGCA
*Gapdh*		CGACTTCAACAGCAACTCCCACTCTTCC	TGGGTGGTCCAGGGTTTCTTACTCCTT
*Tbp*		TGCACAGGAGCCAAGAGTGAA	CACATCACAGCTCCCCACCA
*Slc1a2*		ACAATATGCCCAAGCAGGTAGA	CTTTGGCTCATCGGAGCTGA
*Naca*		GACAGTGATGAGTCAGTACCAGA	TGCTTGGCTTTACTAACAGGTTC

## Data Availability

Data is contained within the article and supplementary material.

## Supplementary Materials

The following supporting information can be downloaded at: https://www.mdpi.com/article/10.3390/antiox11101897/s1; Figure S1: Analysis of Arg-DPRs on cell cycle and survival of NSC34 cell line, Figure S2: Overexpression of NRF2^ΔETGE^ through lentiviral delivery; Table S1: RT² Profiler™ PCR Array Human Oxidative Stress Plus, PAHS-065Y, Qiagen; Table S2: Complete descriptive statistics and statistical analysis of redox-focused profiling in *C9orf72* ALS-patients vs. healthy controls.
