# Supplementary material for: Dipeptide Repeat Pathology in C9orf72-ALS Is Associated with Redox, Mitochondrial and NRF2 Pathway Imbalance
Source: Antioxidants (Basel). 2022 Sep 25;11(10):1897. doi: 10.3390/antiox11101897 (PMC9598689; doi:10.3390/antiox11101897)
Supplement: Supplementary file 1 [file antioxidants-11-01897-s001.zip › Figure S1 and Figure S2.pdf]

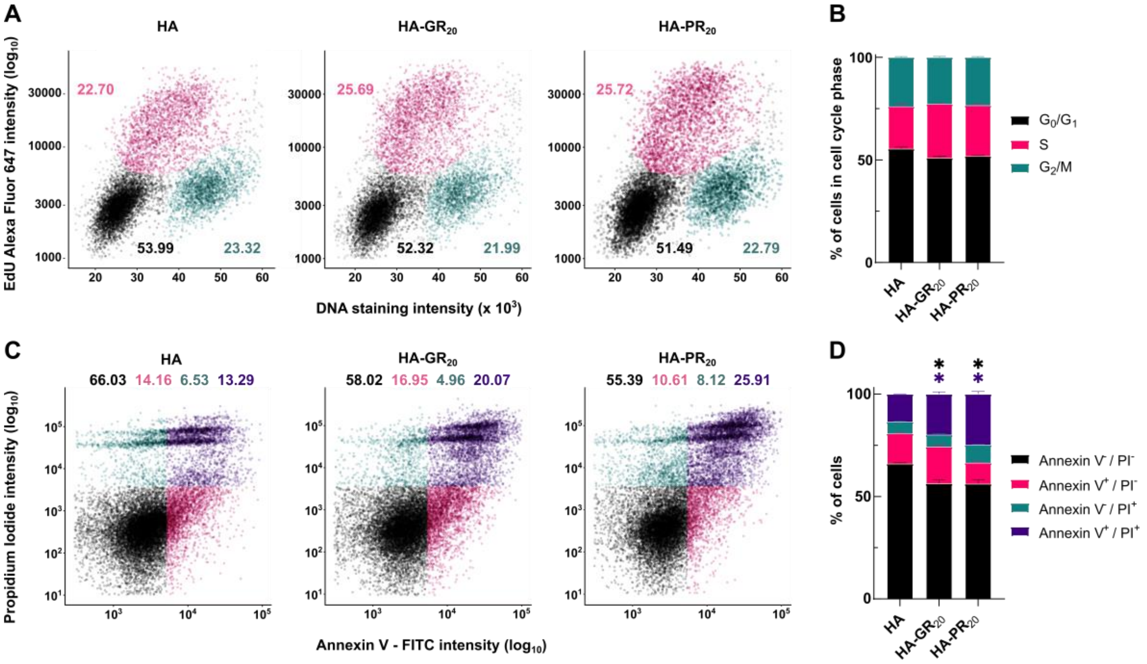

Figure S1. Analysis of Arg-DPRs on cell cycle and survival of NSC34 cell line. A, cell cycle progression measured by flow cytometry of EdU incorporation and DNA content in NSC34 cells treated with either 6 μM HA, HA-GR20 or HA-PR20 for 24h. **A,** representative sample of 10.000 cells is shown for each experimental condition. **B,** comparison of the percentages of G_0/G_1 cells, S and G_2/M determined by EdU and total DNA staining. **C,** determination by flow cytometry of Arg-DPRs-induced apoptosis and necrosis in cells stained with Annexin-V-FITC and propidium iodide (PI). NSC34 cells were treated with either 6 μM HA, HA-GR20 or HA-PR20 for 24 h and then stained for 15 min with 1/100 Annexin-V-FITC and 1 μg/ml PI. A representative sample of 10,000 cells is shown for each experimental condition. **D,** comparison of the percentages of each population,** $p < 0.001$ for cells double-positive and double-negative for Annexin V-FITC and PI comparing Arg-DPRs with HA groups. In A and C, numbers represent the percentage for each population of the shown sample, matching the text colour with the population colour.

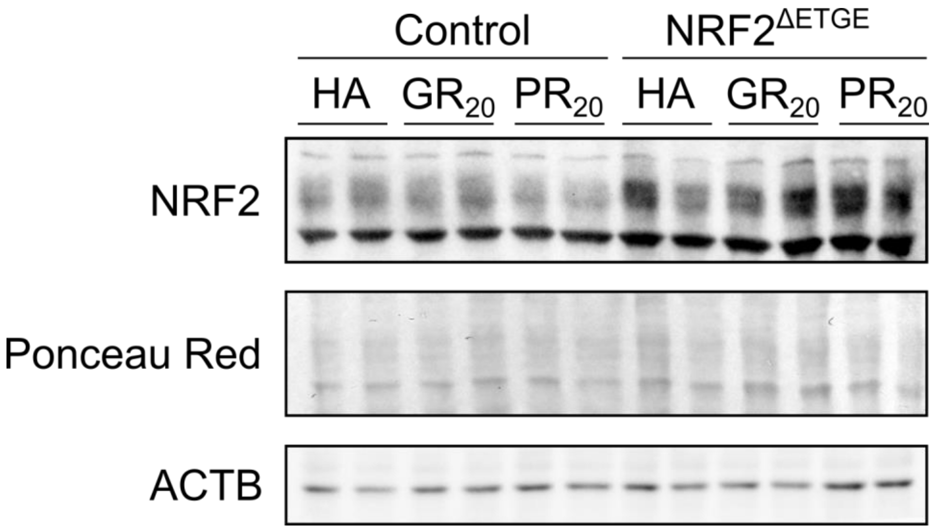

Figure S2 (related to Figure 7). Overexpression of NRF2$^{\Delta ETGE}$ through lentiviral delivery**.** NSC34 cells were transduced with either control lentiviral particles or encoding the stable NRF2 version lacking the ETGE KEAP1-binding motif for 48 h and then treated with 6 μM of HA, HA-GR$_{20}$ or HA-PR$_{20}$ peptides for 24 h. The levels of the indicated proteins were measured by immunoblot. ACTB levels and Ponceau Red staining were determined as loading controls.
