# Supplementary material for: Dipeptide Repeat Pathology in C9orf72-ALS Is Associated with Redox, Mitochondrial and NRF2 Pathway Imbalance
Source: Antioxidants (Basel). 2022 Sep 25;11(10):1897. doi: 10.3390/antiox11101897 (PMC9598689; doi:10.3390/antiox11101897)
Supplement: Supplementary file 1 [file antioxidants-11-01897-s001.zip › Table S1.pdf]

Table S1. RT² Profiler™ PCR Array Human Oxidative Stress Plus, PAHS-065Y, Qiagen

**Endogenous antioxidant systems**	**Metabolism of reactive oxygen species**
Glutathione peroxidases: *GPX1, GPX2, GPX3, GPX4, GPX5, GSTP1, GSTZ1.*<br>***Peroxiredoxins***: *PRDX1, PRDX2, PRDX3, PRDX4, PRDX5, PRDX6 (AOP2).*<br>***Other peroxidases***: *CAT, CYBB, CYGB, DUOX1, DUOX2, EPX, LPO, MPO, PTGS1, PTGS2 (COX2), TPO, TTN.*<br>***Other antioxidants***: *ALB, APOE, GSR, MT3, SELENOS, SOD1, SOD3, SRXN1, TXNRD1, TXNRD2.*	***Superoxide dismutases***: *SOD1, SOD2, SOD3.*<br>***Other genes involved in superoxide metabolism***: *ALOX12, CCS, DUOX1, DUOX2, MT3, NCF1, NCF2, NOS2 (iNOS), NOX4, NOX5, UCP2.*<br>***Other genes involved in ROS metabolism***: *AOX1, BNIP3, EPHX2, MPV17, SFTPD.*<br>***Oxygen transporters***: *CYGB, MB.*
**Oxidative stress responsive genes**	
APOE, ATOX1, CAT, CCL5 (RANTES), CYGB, DHCR24, DUOX1, DUOX2, DUSP1 (PTPN16), EPX, FOXM1, FTH1, GCLC, GCLM, GPX1, GPX2, GPX3, GPX4, GPX5, GSR, GSS, HMOX1, HSPA1A, KRT1, LPO, MBL2, MPO, MSRA, NQO1, NUDT1, PDLIM1, PRDX2, PRDX5, PRDX6 (AOP2), PRNP, RNF7, VIMP, SELENOP, SIRT2, SOD1, SOD2, SQSTM1, SRXN1, TPO, TTN, TXN, TXNRD1, TXNRD2.	
**Pathway activity signature genes**	
*AKR1C2, BAG2, FHL2, GCLM, GLA, HMOX1, HSP90AA1, LHPP, NCOA7, NQO1, PTGR1, SLC7A11, SPINK1, TRAPPC6A, TXN, TXNRD1***.**	
