# Supplementary material for: Dipeptide Repeat Pathology in C9orf72-ALS Is Associated with Redox, Mitochondrial and NRF2 Pathway Imbalance
Source: Antioxidants (Basel). 2022 Sep 25;11(10):1897. doi: 10.3390/antiox11101897 (PMC9598689; doi:10.3390/antiox11101897)
Supplement: Supplementary file 1 [file antioxidants-11-01897-s001.zip › Table S2.pdf]

Table S2. Complete descriptive statistics and statistical analysis of redox-focused profiling in C9orf72 ALS-patients vs. healthy controls.

Gene	Ensembl Gene ID	Mean log_2 FC	SD	Wilcoxon test p-value
DHCR24	ENSG00000116133	-0.72	0.37	0.0023
TRAPPC6A	ENSG00000007255	-0.58	0.27	0.0041
GPX3	ENSG00000211445	-0.59	0.21	0.0041
GSR	ENSG00000104687	-0.44	0.32	0.0070
FHL2	ENSG00000115641	-0.96	0.75	0.0111
CYBB	ENSG00000165168	0.53	0.45	0.0175
NUDT1	ENSG00000106268	-0.35	0.27	0.0262
CCS	ENSG00000173992	-0.67	0.50	0.0262
DUOX2	ENSG00000140279	-1.79	1.63	0.0262
FOXM1	ENSG00000111206	-0.49	0.19	0.0379
PRDX5	ENSG00000126432	-0.20	0.21	0.0728
PRDX6	ENSG00000117592	-0.43	0.24	0.0728
DUOX1	ENSG00000137857	-0.59	0.60	0.0728
PTGS1	ENSG00000095303	-0.53	0.54	0.0973
SIRT2	ENSG00000068903	-0.32	0.39	0.1280
PDLIM1	ENSG00000107438	-0.43	0.51	0.1280
SRXN1	ENSG00000271303	-0.46	0.58	0.1280
UCP2	ENSG00000175567	-0.16	0.22	0.1650
ALOX12	ENSG00000108839	-0.39	0.41	0.1650
CYGB	ENSG00000161544	-0.56	1.12	0.1650
GLA	ENSG00000102393	-0.41	0.45	0.1810
TPO	ENSG00000115705	1.11	2.06	0.2090
LPO	ENSG00000167419	0.52	0.84	0.2400
NQO1	ENSG00000181019	0.19	0.25	0.2590
GPX4	ENSG00000167468	-0.11	0.19	0.2590
RNF7	ENSG00000114125	-0.12	0.21	0.2590
HMOX1	ENSG00000100292	-0.22	0.57	0.2590
SFTPD	ENSG00000133661	-0.39	0.53	0.2590
CAT	ENSG00000121691	-0.42	0.62	0.2590
EPX	ENSG00000121053	-0.50	0.86	0.2590
PRNP	ENSG00000171867	0.22	0.26	0.3180

EPHX2	ENSG00000120915	-0.52	0.77	0.3180
DUSP1	ENSG00000120129	0.21	0.56	0.3830
HSP90AA1	ENSG00000080824	0.14	0.32	0.3830
FTH1	ENSG00000167996	0.12	0.19	0.3830
BAG2	ENSG00000112208	-0.23	0.42	0.3830
NOS2	ENSG00000007171	-0.31	0.66	0.3940
GSTP1	ENSG00000084207	-0.05	0.46	0.4560
MPV17	ENSG00000115204	-0.08	0.17	0.4560
GCLM	ENSG00000023909	-0.09	0.32	0.4560
TXNRD2	ENSG00000184470	-0.13	0.28	0.4560
PRDX2	ENSG00000167815	-0.26	0.52	0.4560
SELENOS	ENSG00000131871	-0.35	0.76	0.4560
SOD2	ENSG00000112096	-0.11	0.37	0.5340
NCOA7	ENSG00000111912	-0.12	0.35	0.5350
PRDX4	ENSG00000123131	-0.17	0.40	0.5350
NCF1	ENSG00000158517	-0.20	0.53	0.5350
SLC7A11	ENSG00000151012	0.09	0.47	0.6200
SOD1	ENSG00000142168	-0.11	0.44	0.6200
TTN	ENSG00000155657	0.18	0.90	0.7100
SELENOP	ENSG00000250722	0.12	0.62	0.7100
LHPP	ENSG00000107902	0.06	0.29	0.7100
PTGR1	ENSG00000106853	0.04	0.75	0.7100
MPO	ENSG00000005381	0.01	1.10	0.7100
GPX1	ENSG00000233276	-0.04	0.32	0.7100
KRT1	ENSG00000167768	-1.01	4.50	0.7100
MSRA	ENSG00000175806	0.05	0.32	0.8050
ATOX1	ENSG00000177556	-0.01	0.21	0.8050
TXNRD1	ENSG00000198431	-0.05	0.25	0.8050
BNIP3	ENSG00000176171	-0.10	0.44	0.8050
HSPA1A	ENSG00000204389	-0.10	0.60	0.8050
CCL5	ENSG00000271503	-0.20	0.91	0.8050
AKR1C2	ENSG00000151632	-0.25	2.10	0.8050
GCLC	ENSG00000001084	0.30	0.59	0.9020

PRDX1	ENSG00000117450	0.05	0.51	0.9020
GSS	ENSG00000100983	-0.03	0.27	0.9020
GPX2	ENSG00000176153	-0.05	0.36	0.9020
NCF2	ENSG00000116701	-0.08	0.27	0.9020
PTGS2	ENSG00000073756	-0.09	0.90	0.9020
TXN	ENSG00000136810	0.01	0.28	1.0000
GSTZ1	ENSG00000100577	-0.04	0.32	1.0000
SQSTM1	ENSG00000161011	-0.05	0.29	1.0000
PRDX3	ENSG00000165672	-0.07	0.51	1.0000
